# Inhibiting Autophagy by Chemicals During SCAPs Osteodifferentiation Elicits Disorganized Mineralization, While the Knock-Out of *Atg5/7* Genes Leads to Cell Adaptation

**DOI:** 10.3390/cells14020146

**Published:** 2025-01-20

**Authors:** Damien Le Nihouannen, Claudine Boiziau, Sylvie Rey, Nicole Agadzhanian, Nathalie Dusserre, Fabrice Cordelières, Muriel Priault, Helene Boeuf

**Affiliations:** 1 Univ. Bordeaux, INSERM, BIOTIS, U1026, F-33000 Bordeaux, France; damien.le-nihouannen@u-bordeaux.fr (D.L.N.); sylvie.rey@inserm.fr (S.R.); nikol.agadzhanian@yale.edu (N.A.); nathalie.dusserre@inserm.fr (N.D.); 2 Bordeaux Imaging Center (BIC), US4, UAR 3420, National Center for Scientific Research (CNRS), National Institute of Health and Medical Research (INSERM), Université de Bordeaux, F-33000 Bordeaux, France; fabrice.cordelieres@u-bordeaux.fr; 3 National Center for Scientific Research (CNRS), Institut de Biochimie et Génétique Cellulaires (IBGC), UMR 5095, Université de Bordeaux, F-33000 Bordeaux, France; muriel.priault@u-bordeaux.fr

**Keywords:** mesenchymal stem cells, SCAPs, canonical autophagy, alternative autophagy, bafilomycin A1, verteporfin, LC3, ALP activity, osteoblasts, mineralization

## Abstract

SCAPs (Stem Cells from Apical Papilla), derived from the apex of forming wisdom teeth, extracted from teenagers for orthodontic reasons, belong to the MSCs (Mesenchymal Stromal Cells) family. They have multipotent differentiation capabilities and are a potentially powerful model for investigating strategies of clinical cell therapies. Since autophagy—a regulated self-eating process—was proposed to be essential in osteogenesis, we investigated its involvement in the SCAP model. By using a combination of chemical and genetic approaches to inhibit autophagy, we studied early and late events of osteoblastic differentiation. We showed that blocking the formation of autophagosomes with verteporfin did not induce a dramatic alteration in early osteoblastic differentiation monitored by ALP (alkaline phosphatase) activity. However, blocking the autophagy flux with bafilomycin A1 led to ALP repression. Strikingly, the mineralization process was observed with both compounds, with calcium phosphate (CaP) nodules that remained inside cells under bafilomycin A1 treatment and numerous but smaller CaP nodules after verteporfin treatment. In contrast, deletion of *Atg5* or *Atg7*, two genes involved in the formation of autophagosomes and essential to trigger canonical autophagy, indicated that both genes could be involved differently in the mineralization process with a modification of the ALP activity while final mineralization was not altered.

## 1. Introduction

Tissue engineering for bone regeneration aims at identifying cells that could promote efficient osteogenesis. Mesenchymal stromal cells (MSCs) of different origins have long been exploited in a research context, of which the multipotent SCAP cells (Stem Cells from Apical Papilla), isolated from the apical papilla of wisdom teeth, may provide effective solutions.

The apical papilla tissue, the vascularized cushion that lies between the teeth roots in formation and the jaw bone, is located at the apex of the extracted teeth. Like the bone marrow from which MSCs are classically derived, wisdom teeth are a surgical waste, and there are no ethical restrictions regarding the potential clinical use of SCAPs, except the donors’ agreement.

SCAPs are preferentially differentiated towards mesodermal lineages (osteoblasts, adipocytes, and chondrocytes) but are also prone to differentiate into neuronal cells in link with their embryonic neural crest origin [1,2,3,4]. In the context of bone regeneration, SCAPs may be used to promote matrix mineralization, as already suggested by other studies using these cells [5,6,7] or other MSCs and mice models [8,9,10,11]. The mineralization regulation during the osteogenic differentiation process is, therefore, of great interest and was dissected in various cell models. Among the cell regulatory pathways, autophagy was described as being essential for proper osteogenic differentiation [12,13,14]. However, how autophagy contributes to osteogenic differentiation remains unclear. Is it involved, for example, in the protected transport of calcium phosphate-containing vesicles outside cells? Could blocking the autophagy flux alter specifically the secretion of compounds essential for the formation of a mineralized matrix? Could cells adapt when canonical autophagy is absent?

Autophagy is a highly regulated self-eating process that contributes to removing unnecessary or dysfunctional cellular components through a controlled pathway of degradation and recycling [15,16]. This process can be induced by many stresses like nutrient starvation or sudden hypoxic switch. It allows the engulfment of cytoplasmic elements (unfolded proteins, RNAs, mitochondria, etc.) in double-membrane-bound vesicles called autophagosomes. These vesicles are formed in the cytosol upon the activation of a cascade of autophagy-regulated (*Atg*) genes. The ATG7 and ATG10 enzymes mediate the covalent conjugation of the ubiquitin-like ATG12 to ATG5, which then binds to ATG16, an essential step in autophagosome formation [10]. ATG7 is further required, with ATG3, to conjugate PE (phosphatidyl ethanolamine) to the microtubule-associated proteins of the Light Chain 3 (LC3) family (e.g., LC3, GABARAP, GATE16). These proteins decorate autophagosomes and interact with molecular motors for transport along microtubules. Autophagosomal cargo is thus conveyed to lysosomes for degradation [17,18,19] or secretion [20]. After degradation, transporters of the autolysosomal membrane release molecular building blocks that are available to sustain the viability of stressed cells or simply help the controlled recirculation of digested metabolites. Autophagy is considered an internal quality control of cells and can be modulated by inhibitory or activating chemical compounds. Thus, autophagy is exquisitely tractable, and various effects may be monitored depending on cell types and cell metabolism. For example, rapamycin is a potent inducer of autophagy since it blocks mTOR (mechanistic target of rapamycin), an autophagy repressor. This compound has been extensively used to induce autophagy in different cell models [21,22]. However, complex effects of rapamycin on osteoblast differentiation, depending on dose and differentiation kinetics, were reported [14]. Verteporfin, an FDA-approved compound used to alleviate the symptoms of particular eye diseases [23,24,25], has been selected as a potent repressor of autophagosome formation; this compound is also used for its negative effect on the HIPPO/YAP pathway— the two properties are not necessarily linked [26,27,28]. Furthermore, by altering the acidification of lysosomes, compounds like bafilomycin A1 or chloroquine block autophagosome/lysosome fusion and thus prevent the degradation of autophagosomes’ cargo. The resulting blockade of the active autophagy flux can lead to cell death if prolonged. Therefore, autophagy can be altered at different steps by chemicals. This approach is instrumental in deciphering the importance of each player, with the warning that these drugs may also display off-target and toxic effects.

In addition, autophagy has specific functions in embryonic and adult stem cell models [29,30,31,32]. For example, it maintains stemness by preventing senescence in geriatric muscle satellite stem cells [33]. Autophagy has also been shown to be essential in osteogenic differentiation in a model of rat osteosarcoma cell line [11] and in vitamin K2-induced osteogenesis in a murine pre-osteoblast cell line [34]. Disruption of the autophagy process has also been described in osteoporosis, counterbalanced by estradiol or vitamin K2 treatment [34,35].

*Atg5* KO mice die at birth due to neuronal dysfunction, leading to suckling failure. In transgenic mice where ATG5 loss was specifically rescued in the brain to circumvent neuronal dysfunction and death at birth, many organ defects were reported, including bones [36,37]. Specific deletion of *Atg7* in bones attests to its efficient role during osteogenesis in mice models [10,38]. In addition, devastating human diseases are linked with point mutations in the *Atg7* gene [39].

We have previously characterized the stem cell niche of the apical papilla tissue and shown that MSC markers (such as CD105 and CD90), along with particular stemness ones (SSEA4 and CD49f) identified in SCAPs grown under physioxic oxygen concentration (3% O_2_), are expressed in the native tissue [40]. We have derived independent SCAP banks from apical papilla from different teenagers and characterized their properties by following growth curves, marker expression, and differentiation potentials [7,40]. In our previous work, we were interested in deciphering specific properties of SCAPs grown under physioxic (3% O_2_) *versus* hyperoxic conditions (21%, ambient air) and found that SCAPs display a proliferative advantage and could be amplified for more passages under 3% O_2_ versus 21% O_2_. We have also shown that SCAPs display an active autophagy flux at both O_2_ concentrations with an increased flux when SCAPs are switched from 21% to 3% O_2_, which is stabilized after a few days at low O_2_ concentrations [7].

In this study, we concentrated on the impact of constitutive canonical autophagy in the process of osteogenic differentiation and here, we present intriguing results obtained from different SCAP donors and *Atg* KO cell lines grown at 21% O_2_. We showed that transient and repetitive alteration in autophagy with chemicals led to an imperfect mineralization process, while permanent autophagy inhibition through disruption of some *Atg* genes led to cell adaptation, eventually leading to productive differentiation and mineralization.

We showed that transient and repetitive alteration in autophagy with chemicals led to an imperfect mineralization process, while permanent autophagy inhibition through disruption of some *Atg* genes led to cell adaptation, eventually leading to productive differentiation and mineralization.

## 2. Materials and Methods

The study was conducted in accordance with the Declaration of Helsinki and after approval of the French Research Ministry (DC 2008-412). Wisdom teeth were collected at the Centre Hospitalier Universitaire (CHU) of Bordeaux (Groupe Hospitalier Saint André, Bordeaux, France) according to the procedure approved by French regulations. Teeth were collected after informed and oral consent was obtained from the donors and their parents according to the ethical guidelines set by French law.

### 2.1. SCAP Banks

All SCAP banks derivation and cell culture were performed as previously published [7,40], using complete medium: alpha MEM (Minimum Essential Medium) (Corning, 10-009CV, Dominique DUTSCHER SAS, 2C, rue de Bruxelles, 67170 Bernolsheim, France), supplemented with Embryomax nucleosides (Millipore, 100X, ES-008D, Millipore SAS., 39 Route Industrielle de la Hardt, 67120, Molsheim, France), 10% fetal bovine serum (FBS, batch N°: S1900-500, Dominique DUTSCHER SAS) and gentamycin (Gibco, 15750-037, 40 µg/mL, Fisher Scientific SAS—Boulevard Sébastien Brant—F67403 Illkirch Cedex, France).

Banks used in our former study were previously named UBx-SCAP-N1, N3, N4, and N5 (derived and amplified at 5% CO_2_/21% O_2_) [40]. In the current study, and for simplicity, the different banks are named donor N1, donor N3, donor N4, and donor N5. Cells were regularly diluted and seeded on T25 flasks (10,000 cells/cm^2^) when they reached 80% confluency. All experiments were performed with early passages (from P4 up to P10).

### 2.2. Lentiviral Infection for Establishment of the Knock-Out (KO) Cell Lines with CRISPR/CAS9 Tools

250,000 SCAPs from donor N1 (passage P6) were transduced, in suspension, in 2.5 mL of complete medium (in T25 flasks) at an MOI (multiplicity of infection) of 2 with the lentiviruses encoding either a RNA guide for *Atg5*, *Atg7* or a scrambled RNA guide (Lenti Ctr) along with Cas9 and a puromycin cassette [41]. The medium was changed 24 h after infection, and puromycin selection (In vivoGen, N°ant-pr, 5 rue Jean Rodier F-31400 Toulouse, France) at 2 µg/mL was started 5 days after infection. Cells were amplified for a week and processed for protein extraction, immunolabeling, or differentiation procedure. A similar procedure was also performed with SCAPs from donor N3, and similar results were obtained.

### 2.3. Chemical Compound Treatments and Immunolabeling

20,000 SCAPs were seeded in 24-well plates (high resolution plates, IBIDI, Ibitreat, 82406, CliniSciences, 74 rue des Suisses, 92000 Nanterre, France), grown for 4 days in complete medium, treated or not for 5 h with 7 µM verteporfin (Sigma-Aldrich, SML 0534, Sigma Aldrich Chimie S.a.r.l, 80 Rue de Luzais, L’lsle-d’Abeau Chesnes—BP701, 38297 Saint-Quentin-Fallavier Cedex, France) or 0.1 µM bafilomycin A1 (LC laboratories, B1080-5M, Woburn, MA 01801 USA), fixed with 4% PFA in PBS for 15 min at room temperature (RT). Cells were labeled with LC3B antibody after a wash in PBS and one with PBS-0.1% gelatin (Sigma-Aldrich, G1890). Cells were then permeabilized with 50 µg/mL digitonin (Sigma-Aldrich, D6628) in PBS-0.1% gelatin for 10 min at RT. Then, cells were washed in PBS-0.1% gelatin for 5 min on a rocker and then incubated with PBS-0.1% gelatin for 30 min at RT. Cells were incubated with the primary antibody anti-LC3B (dilution 1:150 in PBS-0.1% gelatin) overnight at 4 °C. After one wash in PBS-0.1% gelatin for 5 min, cells were incubated with the secondary antibody (goat anti-mouse-coupled Alexa Fluor 488) (diluted 1:1000) in PBS-0.1% gelatin for 1 h at RT. Then, cells were washed in PBS-0.1% gelatin for 5 min and then twice in PBS for 5 min and counterstained with 1 µg/mL DAPI (Thermo scientific-SG2423831, Fisher Scientific S.A.S., Parc d’innovation, Bd Sébastien Brant, 67403 Illkirch, France). Pictures were taken with the confocal microscope Leica TCS SPE with the Leica CTR6500 Electronic Box. When indicated, cells were immunolabeled with Tomm20 and ALP antibodies with an optimized procedure [42]. Briefly, cells were permeabilized in 0.3% Triton-X100 in PBS for 15 min at RT, washed once with PBS, and incubated with the Blocking Solution (PBS containing 0.1% of BSA (Bovine Serum Albumin, A9418, Sigma-Aldrich), 10% of goat serum, (ab7481, Abcam, ), 0.2% Triton X-100, and 0.05% Tween-20) for 1 h at RT. Primary antibodies, diluted in the Blocking Solution, were incubated with cells overnight at 37 °C. After two washes with PBS, cells were incubated with the secondary antibodies diluted 1:1000 in the Blocking Solution for 2 h at 37 °C, washed with PBS, and counterstained with DAPI, 1 µg/mL, in PBS. For immunolabeling on PFA-fixed cells, the following antibodies were used: LC3B (1:150, MBL: M152-3, clone 4E12); Tomm20 (1:500, (ProteinTech, rabbit polyclonal AB,11802-1-AP, Fisher Scientific SAS., Parc d’innovation, Bd Sébastien Brant, 67403 Illkirch, France); ALP (1:100, Novus biologicals, mouse monoclonal antibody NB110-3638, Bio-techne, 19 rue Louis Delourmel, 35230 Noyal Chatillon sur Seiche, France). Secondary antibodies, diluted 1:1000, were from InvitroGen (Thermo Fisher Scientific, Fisher Scientific S.A.S.); these were goat anti-rabbit conjugated to Alexa Fluor 568 (A-11036) or to Alexa Fluor 488 (A-11008); they were also goat anti-mouse conjugated to Alexa Fluor 568 (A-11031) or to Alexa Fluor 488 (A-11001), depending on the primary antibody used.

### 2.4. Osteogenic Differentiation and Treatments

For osteogenic differentiation, SCAPs from all indicated donors or from the KO *Atg* cell lines were plated in 24-well plates at a density of 5000 cells (for labeling at D7), 1000 cells (for labeling at D14), or 200 cells (for labeling at D21) in 1 mL of control medium (alpha MEM containing 10% FBS). Differentiation was started on the day after plating using the differentiation cell kit medium—the StemPro^®^ Osteocyte/Chondrocyte Differentiation Basal Medium (Gibco—A10069-01, Fisher Scientific SAS.)—with the addition of StemPro^®^ Osteogenesis Supplement (Gibco—A10066-01). Unless otherwise indicated, cells were treated from day 7 of differentiation twice a week for 5 h with 0.1 µM bafilomycin A1 or 2 µM verteporfin in an osteogenic medium. After the treatments, cells were kept in the osteogenic medium up to the next treatment. Activation of the early osteogenic marker alkaline phosphatase (ALP) was revealed with an ALP kit (Sigma-Aldrich, 86R-1KT). Briefly, cells were fixed for 10 min at RT with 2% formaldehyde/0.2% glutaraldehyde in PBS and then processed as described in the kit manual. Picture recording was performed with a microscope (LEICA 3000B using the LAS X software, version 3.7.6.25997). Cell mineralization was revealed with the OsteoImage^TM^ kit (Lonza, N°PA-1503, Ozyme, 17 Avenue de Norvège, 91953 Les Ulis, France) following the supplier procedure or by direct staining with 30 µM calcein (Sigma Aldrich, C0875) for 15 min on cells fixed in 70% ETOH [43]. OsteoImage or calcein staining reagents labeled the hydroxyapatite portion of the mineral nodules (green fluorescence) produced by osteoblasts. Cells were washed with PBS and, when indicated, counterstained with DAPI, 1 µg/mL, in PBS. Pictures were taken with the confocal microscope Leica TCS SPE, software LAS AF, version 2.7.3.9723 equipped with the Leica CTR6500 Electronic Box.

### 2.5. Western Blotting

After 4 days in culture, cell lysates were prepared in RIPA buffer supplemented with protease inhibitor cocktail (Sigma-Aldrich, P8340) and Halt phosphatase inhibitor cocktail (Thermo Scientific, Ref: 78420, 39 rue d’Amagnac quai 8.2, Batiment E2, 33800 Bordeaux, France) —0.5 mL of buffer per T25 flask containing subconfluent cells—after cell scraping and centrifugation for 20 min at 9300 g. Protein concentrations of the clarified lysates were established by the Pierce BCA protein assay kit (Thermo Scientific, 23227). A total of 25 µg of protein lysates were loaded onto a 10% acrylamide/bis-acrylamide protein gel, transferred onto a nitrocellulose membrane with the Turbo transfer apparatus (Biorad; Life Science, 3 Bld Raymond Poincaré, 92430 Marnes-la-Coquette, France), and hybridized with antibodies targeting ATG5 (Cell Signaling Technology, D5G3 Rabbit mAB, Tebubio SAS, 39 rue de Houdan—BP 15, 78612 Le Perray en Yvelines Cedex, France), ATG7 (Cell Signaling Technology, rabbit mABD12B11), or LC3B (Abcam, Rb polyclonal ab51520, 24 rue Louis Blanc, 75010 Paris, France). GAPDH (GeneTex, GT239 mouse monoclonal, GeneTex, Inc., 2456 Alton Pkwy, Irvine, CA 92606, USA) was used as a loading control. Secondary HRP-conjugated antibodies (Jackson ImmunoResearch, goat anti-rabbit IgG, 111-035-045, and goat anti-mouse IgG, 115-035-003, INTERCHIM, 211 bis Avenue JF Kennedy, BP1140, 03103 Montlucon Cedex, France) were used.

### 2.6. Quantification of the ALP Signals

Image segmentation and analysis were performed using the Fiji/ImageJ software, version 2.14.0/1.54g. A macro was written to assist the user. The code and some example images are provided at the following link: https://github.com/fabricecordelieres/IJ-Macro_Assisted-seg-histo-img.

A detailed description of the guided segmentation and analysis steps is provided both on the repository and as Supplemental Material (Figure S1).

### 2.7. Statistical Analysis

The data are presented either as arithmetic means with all the obtained data of the group (Figure 2C) or as arithmetic means ± SD (Figure 5B,C). The differences between the groups were assessed with non-parametric tests using GraphPad Prism 10: Kruskal–Wallis test for more than two independent samples, followed by the Mann–Whitney test for two independent samples. A two-tailed *p*-value less than 0.05 was considered significant.

## 3. Results

### 3.1. Active Autophagy Flux, Based on LC3 Staining, Is Efficiently Blocked with Verteporfin and Bafilomycin A1 Treatments Without Altering Mitochondrial Structures

**Figure 1:**
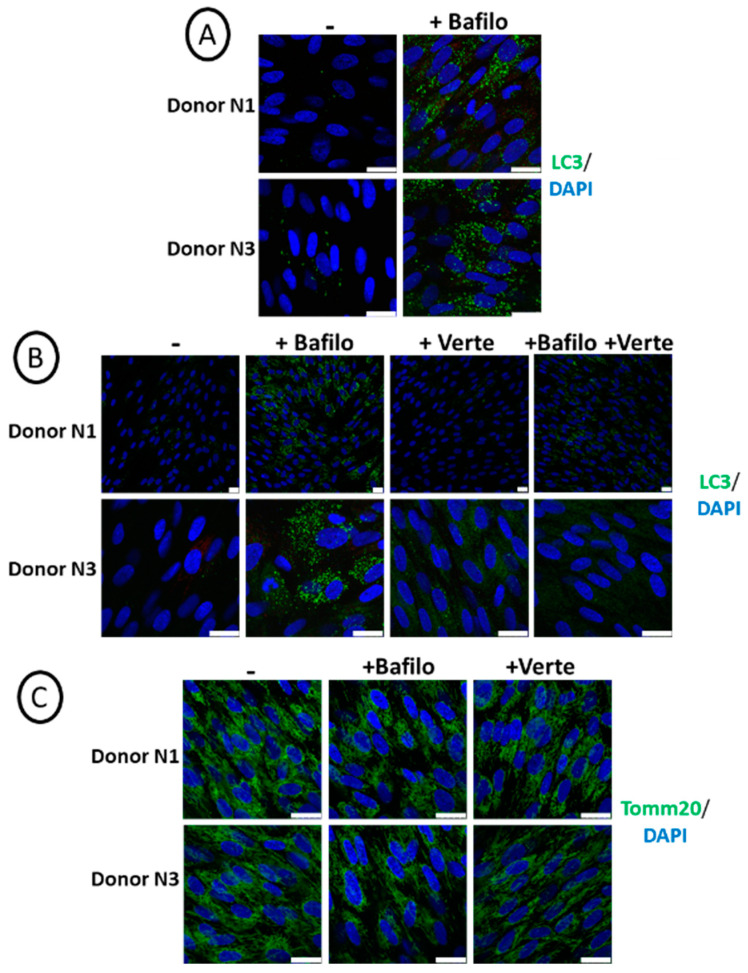
Active autophagy flux in SCAPs, based on LC3 staining. Representative pictures of SCAPs (donors N1 and N3), grown for 4 days in regular cell medium, treated for 5 h before PFA fixation (**A**) with bafilomycin A1 or (**B**,**C**) with bafilomycin A1 (Bafilo) or verteporfin (Verte) or both and immunolabeled with the indicated antibodies. The scale bars are 25 µm.

The overall study aimed to compare the effect of autophagy disruption by chemicals and genetic approaches to better understand its role during osteoblastic differentiation and mineralization. Therefore, we first checked that the early step of autophagy, namely the formation of autophagosomes, was properly repressed by verteporfin. For this purpose, we treated cells grown for 4 days in a regular medium with verteporfin for 5 h. In comparison, bafilomycin A1 was used as a lysosomal inhibitor to block the later steps of autophagosome degradation prior to PFA fixation and immunolabeling with an anti-LC3 antibody. Figure 1A shows efficient autophagy flux in SCAPs based on the detection of LC3 puncta upon bafilomycin A1 treatment. The combined addition of verteporfin and bafilomycin A1 decreases the presence of LC3 puncta, proving that the formation of autophagosomes was impaired by verteporfin treatment (Figure 1B). In addition, short treatment with verteporfin or bafilomycin A1 alone or combined did not have detectable adverse effects on cells since we did not observe apoptotic fragmented nuclei. We also verified that the chemical treatments do not alter mitochondrial structures, as shown by immunolabeling of mitochondria with the anti-translocase of outer mitochondrial membrane 20 (Tomm20) antibody (Figure 1C). This was an important point since chloroquine, a weak base classically used to block autophagosome degradation by alkalinization of lysosomal pH, could impair mitochondrial shape and quality because of its weak base effect [44,45]. This effect was not observed with bafilomycin A1, validating its use in this study.

### 3.2. Blocking Autophagy Flux Alters the Detection of Alkaline Phosphatase Activity While Preventing the Formation of Autophagosomes Is Not Deleterious

The process of osteogenic differentiation of SCAPs can be induced in vitro with a specific osteogenic commercial medium that leads to the formation of osteoblasts, the bone-forming cells. Functional tests allowing the detection of alkaline phosphatase activity (ALP, one of the early markers of osteogenic differentiation) and mineralization (the endpoint function of osteoblasts) can be performed using commercial kits. The osteoblasts synthesize the organic part of the bone matrix (osteoid) and, therefore, mainly secrete type 1 collagen. They also strongly express ALP that hydrolyzes pyrophosphate (PPi) into inorganic phosphate (Pi). Pi associates with calcium to form hydroxyapatite, an essential component of the mineralized matrix. Hydroxyapatite is further detected upon binding to calcein, a green fluorescent component [43,46,47]. The exact role of autophagy in the secretion of calcium phosphate structures (amorphous CaP or hydroxyapatite) formed in the cells in specific vesicles (“matrix vesicles”, MVs [48,49,50,51]) remains an open question. We addressed it by using specific blockers of autophagosome formation (verteporfin) or autophagy flux (bafilomycin A1).

**Figure 2:**
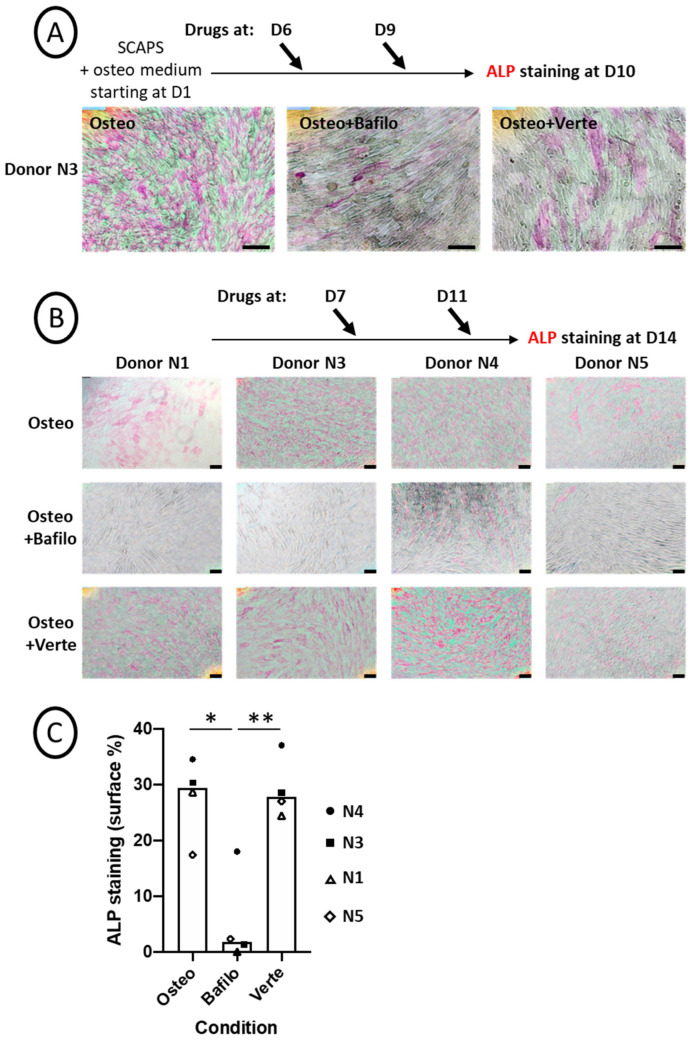
Chemical blockade of autophagy flux alters ALP activity. (**A**,**B**) Representative pictures of ALP activity (pink staining) after ImageJ treatment (the original pictures are shown in Figure S2). SCAP banks were either not treated (Osteo) or treated with bafilomycin A1 (Osteo+Bafilo) or verteporfin (Osteo+Verte) at different time points during the osteodifferentiation process, as indicated. The scale bars are 100 µm. (**C**) Graph of ALP staining quantification (in percentage of stained surface, Figure S1), showing the mean of donors with all independent donors. For each donor, the stained surface was analyzed on one to three fields. Statistics: Kruskal–Wallis test (*p* = 0.013%) and Mann–Whitney (*: *p* = 0.016; **: *p* = 0.009, two-tailed).

SCAP banks (donors N1, N3, N4, and N5) were induced to differentiate into osteogenic medium and treated for 5 h with bafilomycin A1 or with verteporfin at different time points as indicated (Figure 2A,B; original pictures are shown in Figure S2). Cells were processed for ALP activity by a functional test, in which ALP transforms a substrate into a pink product. Repression of ALP activity was observed and quantified for all donors treated with bafilomycin A1. In contrast, verteporfin had almost no effect overall in all the experiments performed (Figure 2C and see Figure S1 for the detailed procedure of quantification). This indicates that while inhibition of autophagosome formation did not disrupt ALP activity, blocking the autophagosomal flux apparently impaired ALP activity, at least the activity our procedure could measure. To rule out the possibility of an artefactual result, we immunolabeled the ALP protein after the staining step. As shown in Figure S3, strong and punctiform labeling of intracellular ALP was evidenced in the Osteo+Bafilo condition; such labeled aggregates were not detected in the Osteo and Osteo+Verte conditions.

### 3.3. Mineralization by SCAPs-Derived Osteoblasts Was Unexpectedly Induced by Bafilomycin A1 or Verteporfin Treatments for Some Donors

We further analyzed the late process of osteoblastic differentiation, which is the formation of the mineralized matrix. Mineralization was revealed by different read-out labelings: either by using the osteoImage kit or by staining with calcein (both giving a green fluorescence staining of the calcium phosphate deposits) or by inducing a brown/red coloration of the deposits with the alizarin red staining.

**Figure 3:**
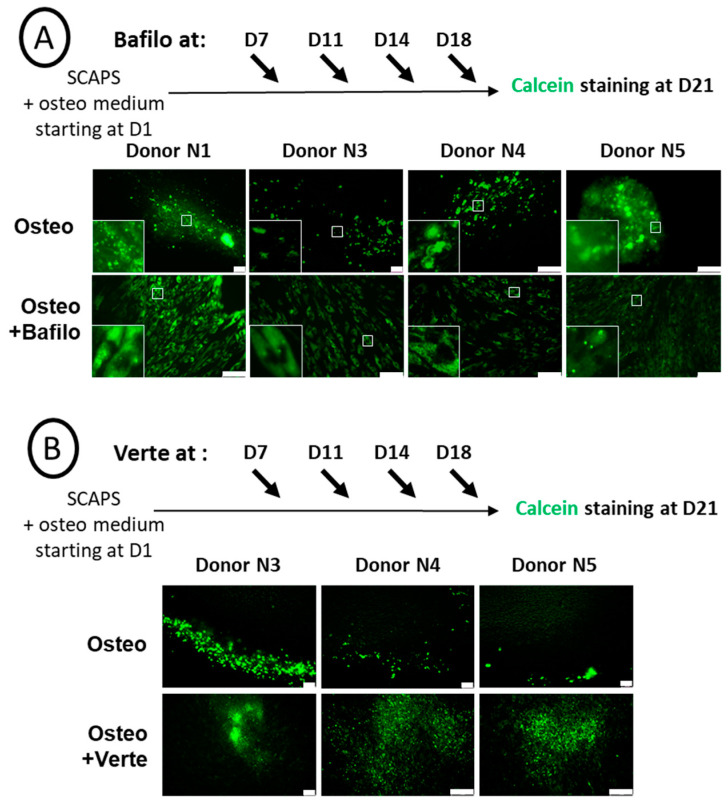
Chemical blockade of autophagy has a donor-dependent effect on mineralization. Representative pictures of calcein staining of SCAP banks from donors as indicated, either non-treated (Osteo) or treated either with bafilomycin A1 (Osteo+Bafilo) (**A**), with magnified images of the white squares shown in insets, or with verteporfin (Osteo+Verte) (**B**). Treatments were applied at different time points during the osteodifferentiation process, as indicated. The scale bars are 100 µm.

SCAPs were induced to differentiate in the osteogenic medium and, from D7, were treated every 3–4 days for 5 h with bafilomycin A1 (Figure 3A) or verteporfin (Figure 3B), then fixed and labeled with calcein at D21. We observed a donor-dependent effect of the bafilomycin A1 treatment, with various effects depending on the timing of the treatment. Indeed, as shown in Figure 3A, mineral formation was induced for donor N1 with a high concentration of green crystals produced inside the cells, while mineralization in cells from the other donors was much less pronounced. Of note, in the latter, the CaP nodules were detected inside the cells (Figure 3A, insets). In contrast, high secretion of the minerals was observed when bafilomycin A1 treatment was performed twice (instead of four times) and at late time points of osteogenic differentiation (Figure S4). This effect observed with donor N1 could not be attributed to an artefactual effect of the drugs since no green staining was observed in the SCAPs treated with the chemicals in the control alpha MEM medium (Figure S5). Indeed, such an artefact could have occurred if chemicals had induced cell death, leading to the release of ions that would then have precipitated. This is not the case here. In addition, no apoptotic nuclei were observed with these compounds. Disruption of autophagosome formation by verteporfin led to a different behavior: mineralization did occur, but the hydroxyapatite crystals formed, although numerous, were smaller than the ones observed without treatment (Figure 3B). In addition, heterogeneous responses of the donors were again observed; indeed, in contrast with other donors, donor N1 displayed no mineralization activity after the verteporfin treatment (Figure S5).

Altogether, the results obtained using these chemical compounds, and based on LC3 puncta detection, indicate that a transient and repeated repression of autophagy or a blockade of the autophagy flux leads to an abnormal mineralization process.

### 3.4. Analysis of Osteoblastic Differentiation Process with CRISPR/CAS9 Knock-Out Strategy

**Figure 4:**
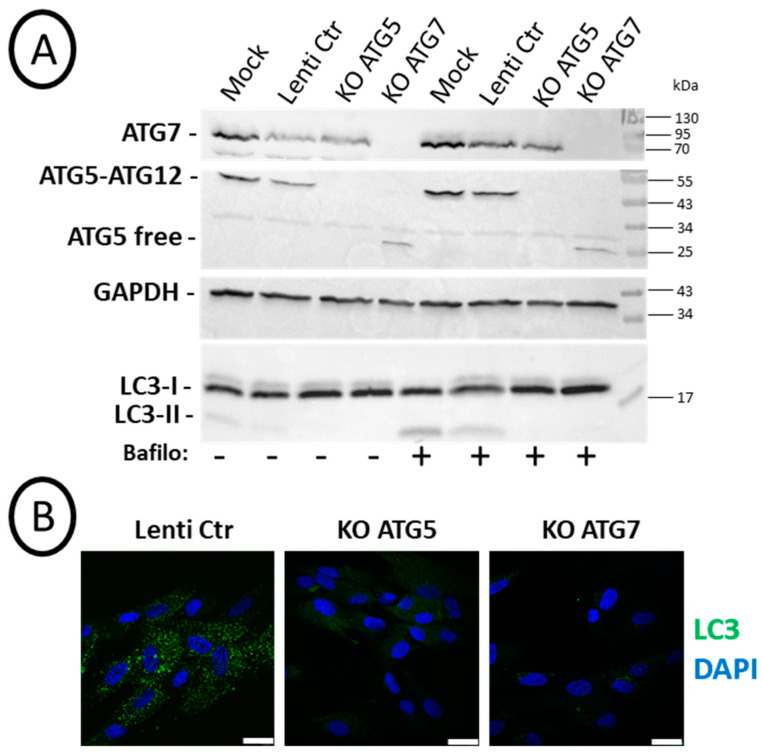
Efficient knock-out (KO) of ATG5 or ATG7 leading to the absence of LC3-II: SCAPs (donor N1) were transduced with different CRISPR/Cas9 lentivirus. (**A**) Western blot analysis of SCAPs treated or not for 2 h with 0.1 µM bafilomycin A1 before cell lysates preparation. Cells non-transduced (Mock), transduced with the control lentivirus (Lenti-Ctr), or with the lentivirus expressing the RNA guide for *Atg5* (KO ATG5) or *Atg7* (KO ATG7) were analyzed with the indicated antibodies. (**B**) Representative pictures of SCAPs treated for 2 h with 0.1 µM bafilomycin A1 before fixation in 4% PFA and immunolabeling with the anti-LC3 antibody. Nuclei were stained with DAPI. The scale bar is 25 µm.

Chemical inhibition of autophagy with bafilomycin A1 or verteporfin led to complex results which could be linked (i) to off-target effects of these compounds and (ii) to the fact that autophagy was only transiently inhibited (5 h every 3/4 days over 2 weeks as shown above). Therefore, we undertook the analyses of cell differentiation with knock-out (KO) SCAP cell lines (donor N1) in which *Atg5* or *Atg*7 were permanently suppressed with the CRISPR/CAS9 strategy. Efficient repression in the expression of these genes was observed in cell lines transduced by lentivirus (Figure 4A). Indeed, western blot analyses showed efficient and specific repression of ATG7 and ATG5–ATG12 complex by the respective RNA guides. Free ATG5 protein was detected, as expected, in cells where ATG7 was suppressed. In addition, the expression of LC3-II, the lipidated form of LC3 present on autophagosome membranes, was clearly increased upon bafilomycin A1 treatment, both in Mock or with the control lentivirus (Lenti-Ctr) cell lines. As expected, it was not the case in the *Atg* KO cell lines. This was a valid demonstration of the efficacy of the CRISPR strategy to block canonical autophagy, at least based on LC3-II detection of autophagosomes (Figure 4A). This was also confirmed by immunolabeling of cells treated with bafilomycin A1 just before fixation. Indeed, we observed almost no LC3 puncta staining in the KO *Atg5* or KO *Atg7* cell lines (Figure 4B). We concluded that the CRISPR/CAS9 KO strategy was efficient in repressing the expression of ATG5 and ATG7 proteins and lipidation of LC3.

### 3.5. Osteoblastic Differentiation of the KO Cell Lines: Unexpected Activation of ALP in ATG5-Deficient SCAPs and Presence of Mineralization in Both ATG5 and ATG7- KO Cell Lines

**Figure 5:**
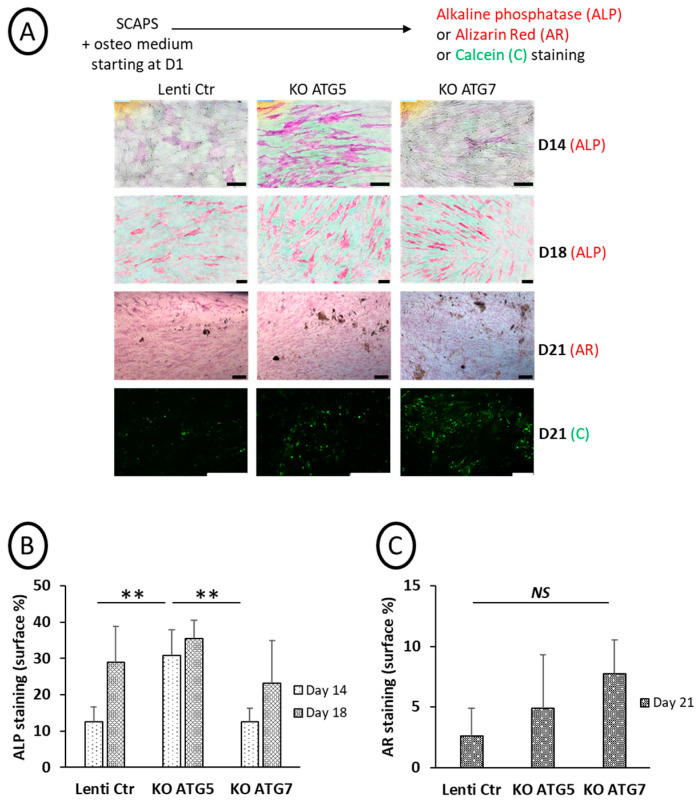
Effects of KO of ATG5 or ATG7 on ALP activity and mineralization. (**A**): Representative pictures of the different SCAP cell lines incubated in an osteogenic medium and processed at different time points for ALP activity (D14 and D18), alizarin red (AR, at D21), or calcein staining (Cal, at D21). The scale bar is 100 µm. Graph of ALP (**B**) and AR signals (**C**) quantification (in percentage of stained surface); mean of five to six fields (**B**) and two to three fields (**C**). Statistics: non-parametric statistical analysis was performed (Kruskal–Wallis and Mann–Whitney tests) to assess the effect of *Atg5* or *Atg7* deletion on ALP activity (KW: *p* = 0.05, MW: **: *p* = 0.006, two-tailed, at D14, non-significant at D18) and on AR staining (non-significant difference at D21). NS: Non Statistical difference.

Osteoblastic differentiation was induced in SCAP cell lines, and ALP activity (at D14 and D18) and mineralization (D21) were analyzed, as shown in Figure 5 (original pictures are displayed in Figure S6). We observed an induction of ALP activity at an early time of differentiation (D14) for the ATG5 KO cell line. At this time point, ALP activity was significantly different in ATG5 KO SCAPs compared to ATG7 or Lenti-Ctr SCAPs (Figure 5B). By contrast, ATG7 KO or Lenti-Ctr SCAPs behaved similarly (Figure 5A,B). However, mineralization occurred in all the cell lines, and important deposits were observed with both Alizarin red and calcein stainings at D21 (Figure 5A,C).

Altogether, these results confirm that disrupting autophagy, at least based on LC3-puncta assays, was not deleterious for mineralization by osteoblasts derived from SCAPs. However, the early stage of osteoblastic differentiation, as assessed by ALP activity, was altered, mainly in ATG5-deficient cells.

## 4. Discussion

Autophagy has been shown to be essential for osteoblastic differentiation on different cellular models, such as immortalized murine MSC lines or dental human MSCs [11,34,35,52]. It has also been shown to be tightly regulated, and an increased autophagy flux has been documented during the first days of the differentiation process, followed by a progressive decrease [14]. Therefore, we investigated its role in mineralization, observed the effects of transient and repetitive inhibition by chemicals, and compared them with permanent genetic inhibition in our cell model, the human SCAPs. Since autophagy was similarly observed under ambient air and physioxic O_2_ concentration, this present study was conducted under 21% O_2_.

### 4.1. Effect of Chemical Repression of Autophagy on Osteogenic Differentiation

The impact of autophagy regulation on osteogenic differentiation was first investigated with two chemicals that are effective in repressing the formation of autophagosomes (verteporfin) or their degradation (bafilomycin A1), as characterized by the limited number of LC3 puncta observed after treatment (Figure 1B). We noticed different effects for these two chemicals: bafilomycin A1 led to the absence of ALP activity detection in the four donor cell lines, while verteporfin did not have a statistically relevant effect. However, unexpectedly, we observed the formation of a mineralized matrix in the presence of bafilomycin A1. While the bafilomycin A1 effect was in agreement with the literature for ALP repression, its impact on cell mineralization was more complex to understand. Indeed, there is a contradiction between the repression of ALP activity within cells and the persistence of mineralization since ALP is an essential enzyme involved in the mineralization process [53,54]. Therefore, we hypothesized that ALP was blocked inside the cells rather than exported to the plasma membrane, which would prevent its detection by the procedure used in this study. This hypothesis is supported by the mineralization assessment. Indeed, the absence of mineral structures secretion was confirmed by calcein staining: CaP structures were detected exclusively inside the cells, in the cytoplasm (Figure 3A, insets); calcein can indeed associate with calcium inside the cells, in the form of amorphous calcium phosphate [48]. However, when bafilomycin A1 treatment was performed at a later time of differentiation when the mineralization process was well underway, we detected calcium phosphate crystals outside cells and even more in the presence of bafilomycin A1 (Figure S4). Interestingly, this indicates that, depending on the kinetics of treatment of the cells with bafilomycin A1, mineralization may be disrupted, with the presence of vesicles that stagnate in the cytoplasm (treatment at early time points) or abnormally increased (treatment at later time points).

However, with the verteporfin treatment, a completely different behavior was observed: despite a lower level of autophagosome formation, ALP activity (present on the cell membrane) was unchanged compared to the control (Figure 2C). However, the mineralized deposits in the extracellular space, as shown by calcein staining, were smaller (Figure 3B). This is in agreement with lower mineralization kinetics [48] due to limited autophagy levels, which is consistent with other studies [34,35]. Verteporfin, also known as a Hippo/YAP repressor, displayed a particular effect in SCAPs, which was in apparent contradiction with the effect described in osteoblastic differentiation, as reported in other cell models. Indeed, for example, previous work performed in MC3T3-E1 cells shows a positive involvement of the YAP pathway in titanium-nanotube-induced osteogenesis [55]. In addition, it has been shown that the YAP/WNT5A/FZD4 axis was essential to the stretch-induced osteogenic differentiation of human periodontal ligament cells (hPDLCs), which was blocked by verteporfin [56]. However, none of these works considered the effect of verteporfin on basal osteogenic-induced mineralization, which was the purpose of our study. To conclude this part, our work shows that canonical autophagy could be involved in the protected transport of calcium phosphate-containing vesicles (Matrix Vesicles, [48]) outside cells. Indeed, when autophagy flux is repeatedly blocked with bafilomycin A1, the calcium phosphate nodules are stored within cells and cannot be secreted. Our results also show that if the formation of autophagosomes is limited by verteporfin treatment, only smaller crystals can be formed and released.

This is in agreement with previous work in which, by transmission electron microscopy, authors visualized mineral nodules inside autophagosomes and hypothesized that autophagic structures could be used by mineralizing osteoblasts to secrete calcium phosphate crystals [11]. In addition, although neither autophagy nor autophagosomes were mentioned, comparable results were described with bafilomycin A1 and lysosome staining with LysoTracker, where it was shown that lysosome transport was involved in amorphous calcium phosphate secretion [48]. Such publications supported the assumption that the autophagy process is involved in mineralization through autophagosome/autolysosome structures containing and secreting mineral nodules.

In addition, a key message of our study is the highlighting of inter-donor variability, which is overlooked when working with unique immortalized or primary cell lines.

### 4.2. Genetic Repression of Autophagy on Osteogenic Differentiation

Since chemicals have (i) potential side effects independent of autophagy and (ii) do not completely and continuously block autophagy, we also developed a genetic approach and established SCAP cell lines in which *Atg5* or *Atg7* genes were deleted by a CRISPR/CAS9 strategy. Puromycin-resistant cells were obtained in which *Atg* gene expression and canonical autophagy (based on LC3 immunolabeling and western blot analysis) were properly repressed. With ATG5 and ATG7 KO cell lines, cells have to adapt to survive during the amplification procedure following infection.

An alternative autophagy process, independent of *Atg5/7*, was described in 2009 that can explain cell survival: in the absence of *Atg5/7*, autophagosomes are detected by transmission electronic microscopy (TEM) inside cells, but they are not decorated by lipidated LC3 proteins [57]. Following this seminal discovery, knowledge of the alternative autophagy process has been acquired [58,59,60]. For example, it was recently demonstrated that NLRP3 inflammasome activation is regulated by alternative autophagy in keratinocytes [61]. The intracellular pathways differentiating canonical autophagy from its alternative counterpart begin to be elucidated: in the context of canonical autophagy, autophagosomes are shown to originate mainly from endoplasmic reticulum or mitochondrial membranes [62], whereas they originate mainly from Golgi membranes in alternative autophagy and this later process was described to be associated with the protein Wipi3 [63]. We suggest that the treatment of ATG 5/7 KO cells with chemical inhibitors such as verteporphin and bafilomycin A1 might help characterize the specificity of alternative autophagy compared with the canonical process.

In our KO cell lines, ALP activity was not inhibited, and mineral deposition in the extracellular matrix was unaffected, either in terms of mineralized surface or nodule size, despite canonical autophagy being prevented. These results suggest that alternative autophagy used by KO cells to survive might promote the formation of autophagosomes that are efficient actors in CaP secretion. However, the potential role of alternative autophagy in mineral nodule secretion remains to be characterized in more detail.

## 5. Conclusions 

In conclusion, the two situations described in this article, transient inhibition of canonical autophagy (by chemicals) and potential use of alternative autophagy (in the KO cell lines), suggest that the CaP nodules (either amorphous or crystals) formed inside matrix vesicles, can be secreted in the extracellular space by both autophagy pathways. Secretory autophagy is emerging as a new research field: autophagy machinery is demonstrated to be involved in the secretion of factors as diverse as cytokines (with the well-described case of IL-1beta [64], granule content (such as von Willebrand Factor from endothelial cells) or lysosomal enzymes from osteoclasts, or extracellular components (reviewed in [20]). Based on our results, we suggest that alternative autophagy might be an actor of secretory autophagy.

The tools developed in our study could help further decipher the mechanism of secretion of mineralized compounds during osteogenesis and pave the way to study new pathways involved in osteogenic differentiation, in link with autophagy. Indeed, the precise mechanisms of extracellular matrix mineralization remain incompletely understood [65]. In the future, it will also be challenging to determine how the autophagy process regulates osteogenic differentiation in KO cells grown under physioxic O_2_ concentration.

## Data Availability

The original contributions presented in this study are included in the article/Supplementary Materials. Further inquiries can be directed to the corresponding author(s).

## Supplementary Materials

The following supporting information can be downloaded at https://www.mdpi.com/article/10.3390/cells14020146/s1. Figure S1: Detailed procedure for ALP quantification. Figure S2: Original pictures of Figure 2 before ImageJ processing for quantification of the stained surfaces. Figure S3: Immunolabeling of ALP: ALP (green) was detected on fixed cells. Cell nuclei were counterstained with DAPI (blue). ALP staining was clearly detectable in the cytoplasm of cells treated with bafilomycin A1; the merged picture (DAPI/ALP+brightfield) is shown for this condition. Fields with cells that were not confluent were chosen to show the intracellular localization of ALP staining. The scale bar is 50 µm. The absence of ALP staining in Osteo and Osteo+Verte conditions was probably due to the procedure used to stain intracellular proteins rather than membrane-associated proteins (Smyreck procedure, [42]) since the activity of the ALP was clearly observed in these conditions. Figure S4: Blocking the autophagy flux at late time points of osteogenic differentiation and just twice along the differentiation process increases the mineralization process for donor N1: Representative pictures of differentiated N1 donor cells labeled with osteoImage kit (green fluorescent hydroxyapatite staining/nuclei in blue) at D20 of the differentiation process, after 5 h of bafilomycin A1 treatment conducted at D10 and D17 (left pictures), or labeled at D23 of the differentiation process, after 5 h of bafilomycin A1 treatment performed at D16 and D19 (right pictures) as indicated by arrows. Two independent experiments are shown, * and **. Figure S5: Chemical treatments did not induce artefactual precipitation of calcium phosphate: SCAPs (donor N1) in alpha MEM medium, (**A**) or in osteogenic medium, (**B**), treated for 5 h with chemicals, at D16 and D19, and labeled with osteoImage kit at D23. These controls were performed in the same experiment as in Figure S4. Figure S6: Effects of KO of ATG5 or ATG7 on ALP activity and mineralization. Original pictures of Figure 5 before image processing for quantification of the stained surfaces.
