# Supplementary material for: Inhibiting Autophagy by Chemicals During SCAPs Osteodifferentiation Elicits Disorganized Mineralization, While the Knock-Out of Atg5/7 Genes Leads to Cell Adaptation
Source: Cells. 2025 Jan 20;14(2):146. doi: 10.3390/cells14020146 (PMC11840282; doi:10.3390/cells14020146)
Supplement: Supplementary file 1 [file cells-14-00146-s001.zip › LENIHO~2.PDF]

Supplemental material

Figure S1

Assisted segmentation of histological images

How does it work ?

1. When the macro is started, an image is assumed to be open. All images except the active image are closed and the ROI Manager, if present, is emptied.
2. The areas corresponding to the data label (top right) and the scale bar (bottom right) are filled in white to avoid these elements being detected as cells.
3. A duplicate of the image is created and named 'Background'.
4. The image is subjected to a large Gaussian filter (sigma: 150 pixels): it is an estimator of illumination inhomogeneity.
5. The original image and the background image are transtyped from RGB to RGB stack. This operation allows the same operation to be performed simultaneously on all 3 channels.
6. The original image (its channels) is divided into the background image. As the result is in 32-bit format to preserve the precision of the calculation, it is renamed to NOM-ORIGINAL_IllumCorr and transtyped to 8-bit. This last step prepares the image for return in RGB (RGB = 3 8-bit channels). The original image is also re-typed in RGB.
7. A background correction using the rolling-ball method is applied to the image corrected for illumination (radius: 50 pixels) and then subjected to a median filter (radius: 5 pixels) to reduce the noise contribution.
8. A dialogue box appears, prompting the user to manually adjust the hue/saturation thresholds in order to identify the pixels carrying the marking.
9. A particle analysis is launched (filter size: 1000 pixels-infinite), resulting in a mask, the ROIs stored in the ROI Manager and a results table (Summary). The mask can be used if detections were to be missed. In this case:
    1. Use a selection tool to crop the cell(s) on the original image.
    2. Recall the selection on the mask: activate the mask image then select Edit/Selection/Restore selection.
    3. Fill the selection with black (double-click on the dropper in the toolbar, then reset the foreground/background colours and select Image/Fill).
10. The original image is activated and the ROIs are shown. A new image is created where the ROIs are drawn on the image copy (Flatten).

A dialog box appears allowing the user to specify the values used for segmentation. These values are logged in the 'Summary' ta

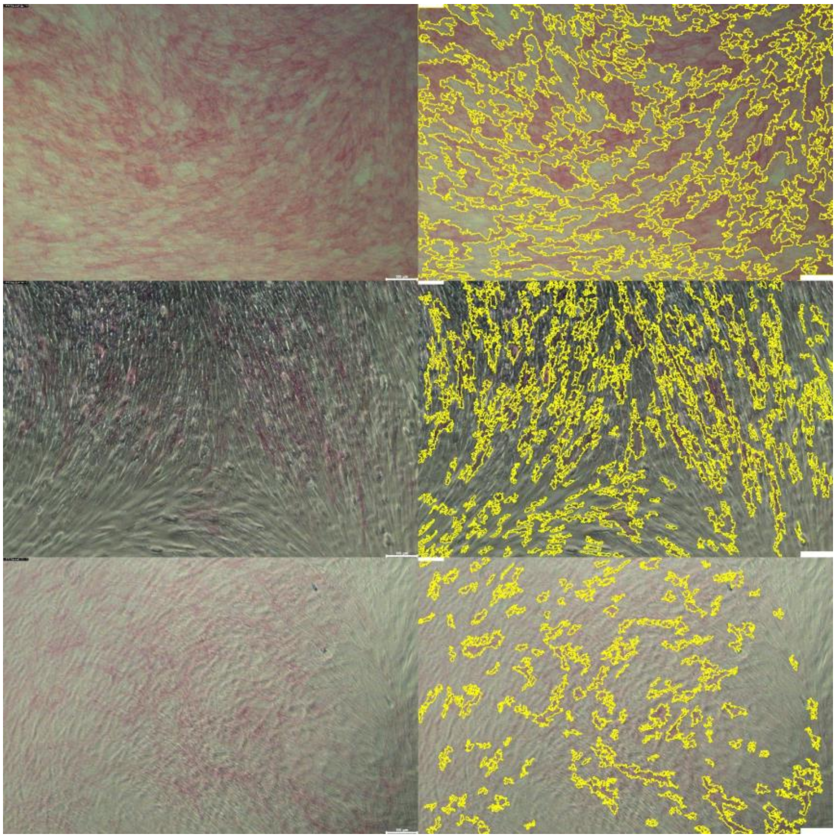

Example of results

How to use it ?

1. Drag and drop the macro file onto the ImageJ toolbar.
2. Open the image to be analysed.
3. In the macro window, select the Macro/Run macro menu.

Versions of the software used: Fiji, ImageJ 2.14.0/1.54f

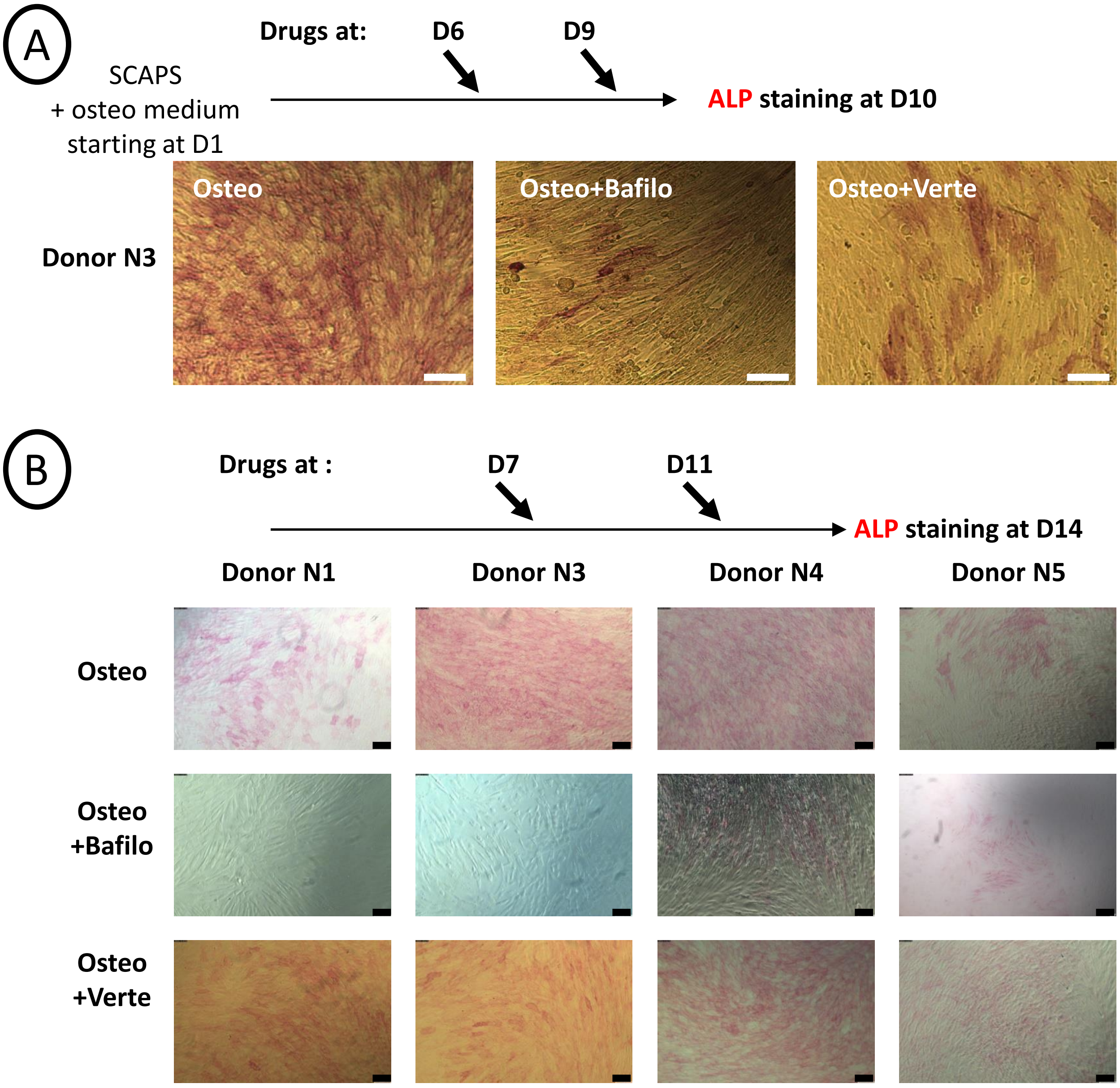

Figure S2. Original pictures of Figure 2 before ImageJ treatment for quantification of the stained surfaces.

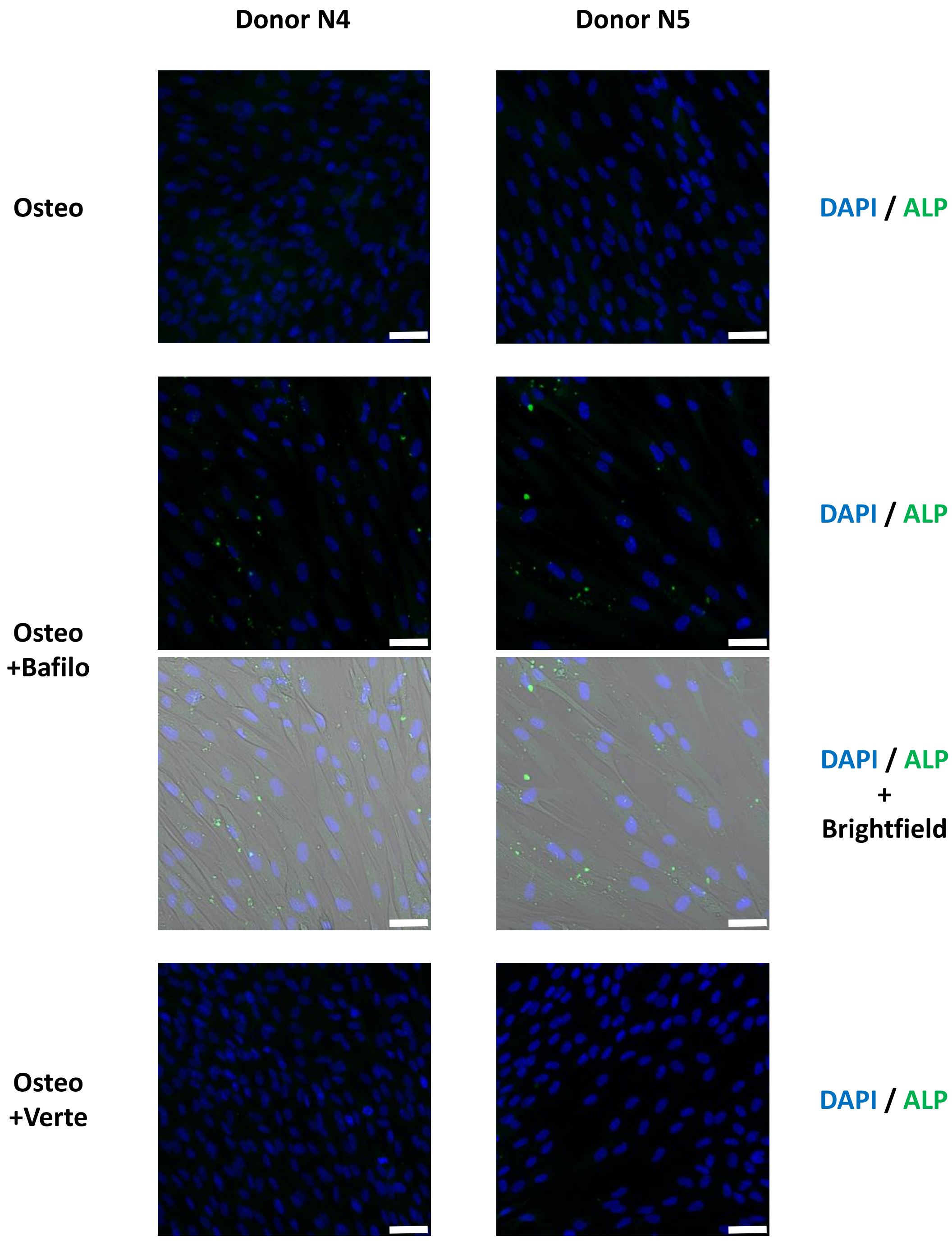

Figure S3. Immunolabeling of ALP.

ALP (green) was detected on fixed cells. Cell nuclei were counter stained with DAPI (blue). ALP staining was clearly detectable in cytoplasm of cells treated with Bafilomycin A1: the merged picture (DAPI/ALP + brightfield) is shown for this condition; fields where cells were not confluent were chosen to show the intracellular localization of ALP staining. Scale bar is 50 μm.

The absence of ALP staining in Osteo and Osteo + Verte conditions was probably due to the procedure used to stain intra-cellular proteins rather than membrane-associated proteins (Smyreck procedure), since activity of the ALP was clearly observed in these conditions.

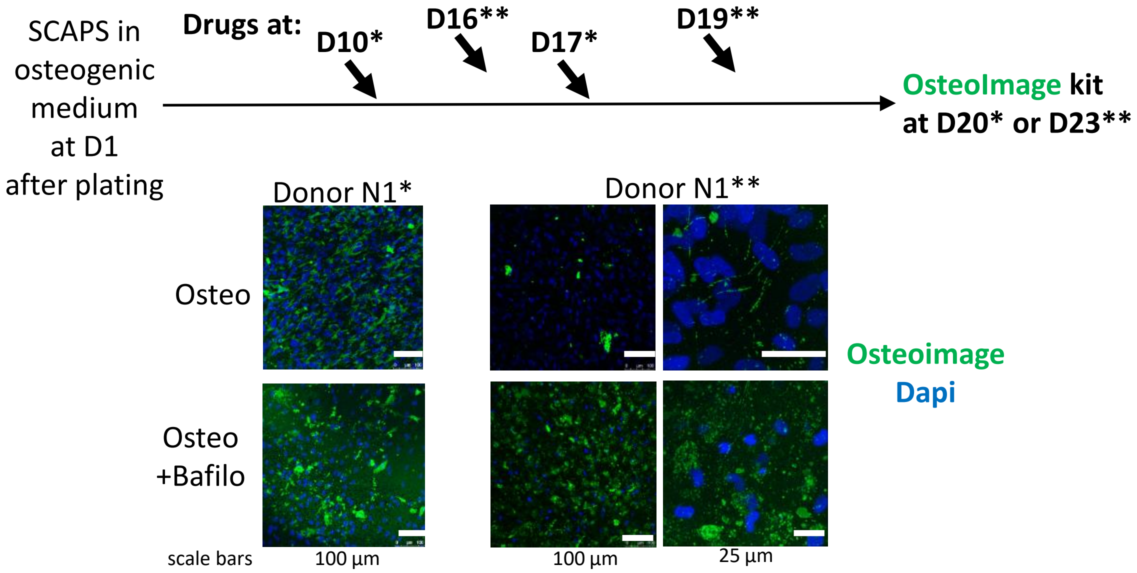

Figure S4: : Blocking the autophagy flux, at late time points of osteogenic differentiation, and just twice along the differentiation process increases mineralisation process on donor N1: Representative pictures of differentiated N1 donor cells labelled with osteoImage kit (green fluorescent hydroxylapatite staining/ nuclei in blue) at day 20 of differentiation process, after 5h of bafilomycin treatment done at D10 and D17, or labelled at day 23 of differentiation process, after 5h of bafilomycin treatment done at D16 and D19 as indicated by arrows. Two independent experiments are shown, * and **.

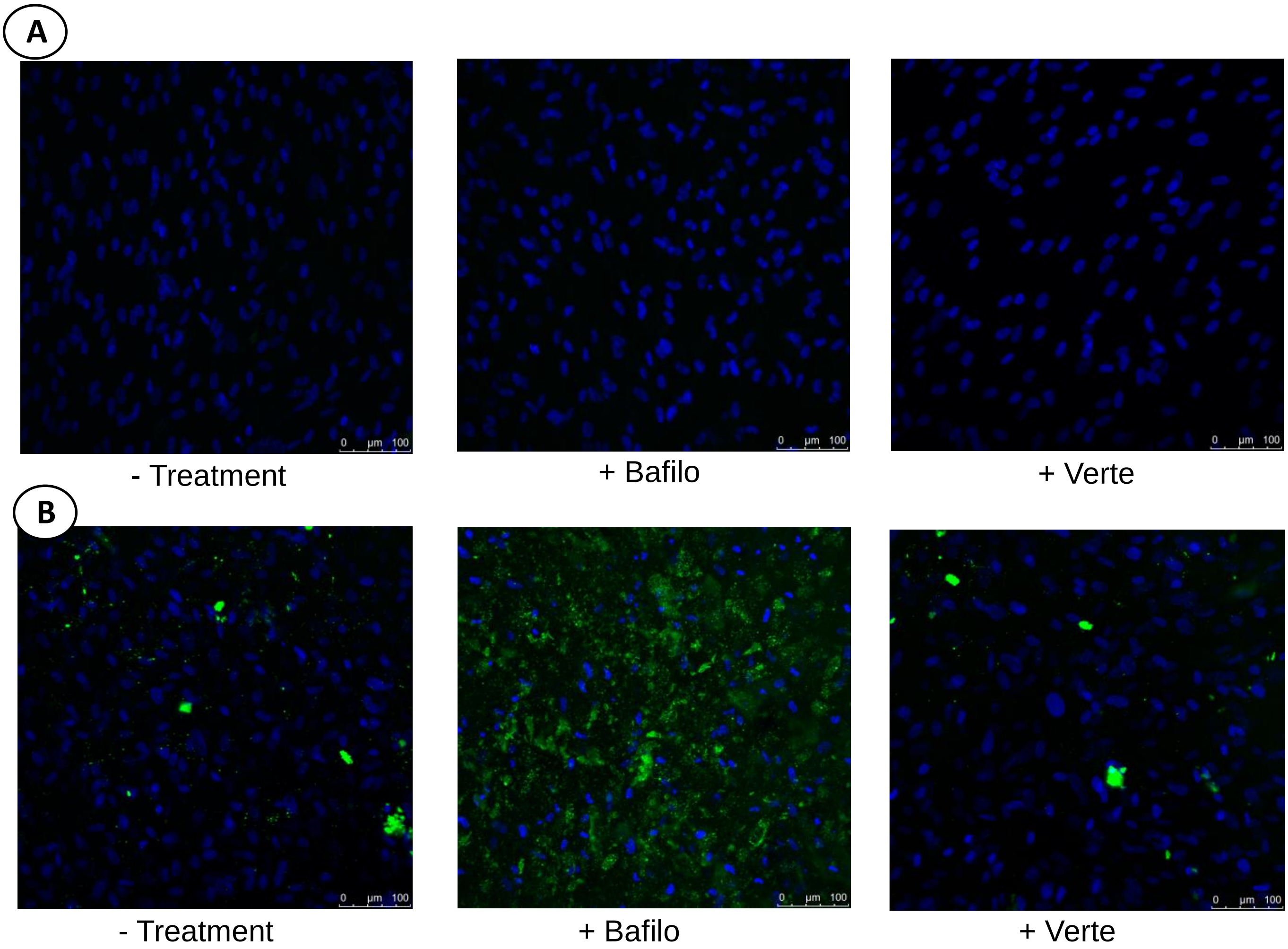

Figure S5 Chemical treatments did not induce artefactual precipitation of calcium phosphate: SCAPs (donor N1) in alpha MEM medium, **A)** or in osteogenic medium, **B)**, treated 5h with chemicals, as indicated, at day 16 and day 19, and labelled with osteoImage kit at D23. These controls have been performed in the same experiment that part of Figure S4.

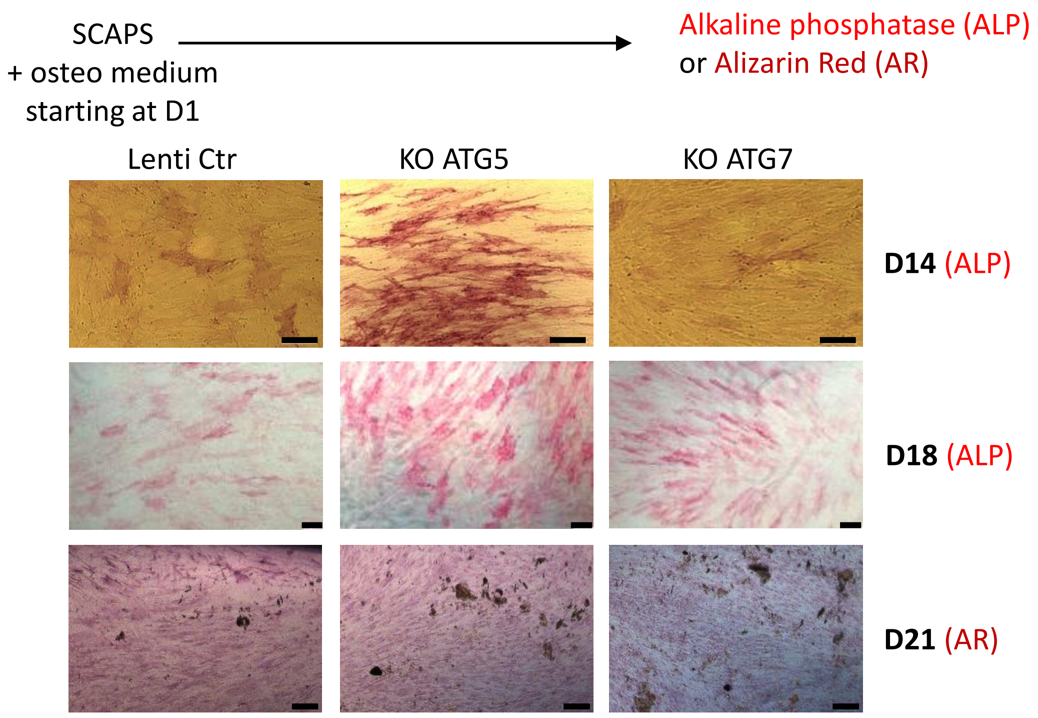

Figure S6. Original pictures of Figure 5 before treatment for quantification of the stained surfaces.
